# Phytochemical Molluscicides and Schistosomiasis: What We Know and What We Still Need to Learn

**DOI:** 10.3390/vetsci5040094

**Published:** 2018-11-06

**Authors:** Ronaldo de Carvalho Augusto, Clelia Christina Correa de Mello-Silva

**Affiliations:** 1 Laboratory of Ecology and Evolution of Interactions 2EI, University Perpignan Via Domitia, IHPE UMR 5244, CNRS, IFREMER, Univ. Montpellier, 66860 Perpignan, France; 2 Laboratório de Avaliação e Promoção e Saúde Ambiental, Oswaldo Cruz Institute, Rio de Janeiro 21040-360, Brazil; clelia@ioc.fiocruz.br

**Keywords:** schistosomiasis, molluscicide, control programs, transmission control

## Abstract

Worldwide schistosomiasis remains a serious public health problem with approximately 67 million people infected and 200 million at risk of infection from inhabiting or transiting endemically active regions. Africa, South America, the Caribbean, and the Middle East are the main transmission regions of *Schistosoma mansoni*. The fight against transmission through the use of molluscicides is not recent and has been advocated as the only activity with the possibility of interruption of transmission in small, epidemiologically active outbreaks. *Euphorbia milii* var. *hislopii* (syn. *splendens*) (Des Moulins, 1826) is the most promising for use in official schistosomiasis control programs according to the WHO. In this review, we show that an understanding of some how *E. milii* latex affects the snail vector and their parasites from a molecular level to field conditions is lacking. On the other hand, this type of treatment could also provide a rationale for the control of schistosomiasis and other parasitosis. Several publications contribute to enforcing the use of *E. milii* latex in endemic countries as a cheap alternative or complement to mass drug treatment with praziquantel, the only available drug to cure the patients (without preventing re-infection).

## 1. Introduction

Worldwide schistosomiasis caused by *S. mansoni* remains a serious public health problem with approximately 67 million people infected and 200 million at risk of infection from inhabiting or transiting endemically active regions [1]. Africa, South America, the Caribbean, and the Middle East are the main transmission regions of *S. mansoni* [2]. Mortality resulting exclusively from *S. mansoni* infection is low, but this pathology presents the highest degree of socioeconomic impact of human helminthiasis [3]. According to Berquist et al. [4], the analysis of the degree of impact of a certain disease that produces a chronic state must be performed in addition to the prevalence and mortality data, so the World Health Organization adopted the Lost Years of Life Disability-Adjusted Life Years (DALY) to measure the degree of impact of endemics that produce chronic status. Based on the DALY, it is estimated that the impact of schistosomiasis can reach 5.8 million years of lost life, with helminth diseases having the greatest impact in the world [4,5].

For the control of schistosomiasis, World Health Organization (WHO) recommends the mass administration of the chemotherapeutic praziquantel (PZQ), control of snails, health education, and technical cooperation between health institutions and research to verify the elimination of infection [6]. The complexity required for control agrees with the complexity of the *S. mansoni* life cycle, and although traditional models based exclusively on the use of schistosomicidal drugs in proven infected individuals have a significant impact on morbidity, the prevalence remains high after decades of treatment. In addition, the appearance of resistance in both laboratory and wild-type lines is an important factor in the search for and development of schistosomicidal compounds. Currently, the only compound used in humans for control and treatment of schistosomiasis is PZQ. Other schistosomicides such as niridazol, hicantone, and oxaminiquine were used in the last century but were abandoned due to their low curative efficiency, host side effects, and/or the appearance of parasitic resistance [7]. Aspects such as oral treatment, low cost, short side effects, and high curative rate are stimulating factors in the use of PZQ as the drug of choice. However, after continuous campaigns of control and mass treatments in Africa and Brazil with this compound, there is a high prevalence and territorial expansion of infection areas [8]. Thus, great attention has been devoted to the discovery of praziquantel-resistant *S. mansoni* strains [9,10,11]. Although the occurrence of resistance is not yet a generalized mechanism among all strains of the parasite [12], this evidence is very worrying and accelerates the need to better understand the mechanisms by which *S. mansoni* has the capacity to support different factors of stress. The rapid adaptability of the parasite to various treatment attempts or precautions deserves special attention from research teams from all over the world [13]. In view of the difficulty of control only with the use of chemotherapeutic agents, WHO encourages other actions such as research and development of vaccine compounds, control of vector snails, health education, and integrated surveillance system.

## 2. Phytochemical Molluscicides and Schistosomiasis: What We Know

The fight against transmission through the use of molluscicides is not recent and has been advocated as the only activity with the possibility of interruption of transmission in small, epidemiologically active outbreaks [14]. The use of molluscicides is generally recommended for rural and urban areas, where the possible environmental damages resulting from the use of these products are not greater than those resulting from human contamination with debris and manure [14].

Controlling or preventing morbidity in patients has not been a very successful strategy to limit schistosomiasis transmission in high-risk areas. Optimal disease prevention can occur only when parasite infection and/or reinfection is effectively impeded [3]. In this sense, the World Health Organization (WHO) published a report of the WHO Strategic and Technical Advisory Group for Neglected Tropical Diseases (NTD) [6]. It addresses schistosomiasis management through the ecological control of population of the intermediate host of the parasite, snails from the *Biomphalaria* and *Bulinus* genus [6]. Molluscicides have been the primary method used for controlling schistosoma transmission. According to the Committee of Experts on Schistosomiasis Control [15], it is well known that projects based only on the use of molluscicidal substances have a clear impact on the incidence and infection of schistosomiasis. This measure was successfully used in the control programs of Ghana, Tanzania, Egypt, and Japan [16]. In other countries, such as Brazil and Zimbabwe, it was possible to observe an intense reduction in the prevalence of infection after the association of chemotherapeutic treatments with the use of molluscicides in their programs [17]. In Zimbabwe, a comprehensive study on toxicity of molluscicide were done and observed a significant toxic effects against aquatic gastropods, fish and amphibians following systematic application of Bayluscide [18]. However, Shiff [19] highlight the risks involved by application of toxic substances in freshwater systems. Although there are no systematic reviews on the actual effects of different methodologies for the application of phytochemical molluscicides, the application in time and not continuous is a consensus among the researchers. The continued use of toxic products in freshwater can cause serious environmental damage beyond what is expected, mainly for non-target organisms.

In this review, molluscicides will be divided into (i) chemical and (ii) phytochemical compounds. Among the chemical compounds, niclosamide (Bayluscide^®^) is recommended by the WHO as the only chemical molluscicide to be used for snail control despite reported cases of resistance in molluscs after two decades of repeated use [20]. Briefly, at recommended doses, Bayluscide^®^ has biocidal activity in non-target organisms such as plants and animals, genotoxicity, and a carcinogenic effect [21]. These factors, associated with the high cost of the product, the possibility of recolonization after treatment interruption, the high ecological toxicity, and the resistance developed by the exclusive use in individuals of the genus *Biomphalaria* and *Bulinus*, are limiting factors for the official use of this compound in large-scale control programs [21].

Nowadays, research and developmental studies on low-dose compounds, biodegradable, low-cost, and less aggressive molluscicidal activity have been intensively investigated [22,23,24]. Among the botanical groups studied, specimens of the families Phytolaccaceae and Euphorbiaceae present great molluscicidal activity for the snail’s intermediate hosts [25,26]. On the one hand, *P. dodecandra* (L’Herit) (synonyms: *P. abyssinica* Hoffm., *Pircunia abyssinica* Moq.), a member of the Phytolaccaceae, is one of the first plants systematically analyzed to be used as a phytochemical molluscicide [27]. The great molluscicidal properties of *P. dodecandra* are related to the high levels of triterpenoids, oleanoic acid, and mainly saponins [26]. In Ethiopia and Zimbabwe, the application of a single dose of *P. dodecandra* berries results in a significant reduction of *Bulinus truncatus* population and, as a consequence, the transmission of schistosomiasis haematobium [28,29,30].

On the other hand, *Euphorbia* genus presents more than 200 species, and it is an important source of medicinal resources for human and veterinary use, as well as in agriculture [31]. In general, they show an abundance of phenolic compounds, as well as groups of secondary metabolites, such as alkaloids, terpenes, glucosinolates, tannins, triterpenes, sesquiterpenes, cerebrosides, glycerols, flavonoids, and steroids, among others [31,32]. Due to its potential medical applications, the genus has been the subject of abundant phytochemical and pharmacological research, highlighting the distinct biological activities described, such as antibacterial action cytotoxic activity, anti-microbial, anti-inflammatory, multidrug resistance modulator, antiviral, gonorrhea, migraine headaches, intestinal parasites, warts, rheumatism, snakebites, and molluscicide, nematocide, among others [33,34,35,36]. However, the use of species of this family is controversial due to the great diversity of secondary metabolites, among them the highly lethal ricin protein present in *Ricinus communis* [37]. Others members of Euphorbiaceae have relevant medicinal compounds used commercially, such as *Euphorbium compositum* from the latex of *Euphorbia resinífera*, a nasal solution indicated for viral infections, rhinitis, and sinusitis, among others, Dysenteral^®^ from the extract of *E. hirta* and indicated for the treatment of diarrheal diseases, and the root extract of *E. kansui* used as a purgative, among many others.

## 3. The Phytochemical Molluscicide *Euphorbia milii* Latex

Of the species belonging to this family, *Euphorbia milii* var. *hislopii* (syn. *splendens*) (Des Moulins, 1826) is the most promising for use in official schistosomiasis control programs according to the WHO [15,38].

*Euphorbia milii* is a shrub with alternating, simple leaves, unisex flowers, gathered in ciliary-like inflorescences with red bracts and campanulate shell with five apical glands [39]. Commonly called the crown-of-Christ, *E. milii* is a shrub originating from Inselberge, located on the central plains of Madagascar (Africa) easily cultivable, as it does not require soil care, fertilizers, and water [40]. In general, latex is an aqueous emulsion found in the vacuole of secretory (latic) cells composed of lipids, resins, sugars, proteins and enzymes [41].

Among the bioactive fractions of *E. milii* latex, eight categories of milliamines were isolated and tested for their molluscicidal activity on *Biomphalaria glabrata*, with L-milliamine being the most efficient, with a lethality of 90% below 0.1 ppm [42]. Singh et al. [23] have described milliamines as serine proteases, and thus receive special attention in industry and pharmaceutical biotechnology based on their property as active on a large scale when at high temperature and pH. Proteases such as eumilin were also isolated from the latex and described as a 30 kDa protein with caseinolytic and fibrinogenolytic activity [43]. A wide range of applications has been reported to *E. milii* latex over the past several years; however, the research of molluscicidal activity was initiated by Vasconcellos & Schall [44] against *B. glabrata* and *B. tenagophila* at concentrations lower than 0.5 ppm. Based on the promising results obtained, the Oswaldo Cruz Institute (FIOCRUZ, Rio de Janeiro, Brazil) deposited the patent for collection, use, and storage in 1998.

The crude extract of *E. milii* is a complex solution and when undiluted can cause inflammatory reaction in the mucous membranes and skin is integrated by the action of phorbol esters and proteases of low pH (4.5 to 5.5), as well as burning in the lips, in the tongue, and in the buccal mucosa in case of ingestion [45]. For use as a molluscicide, many authors focused on the toxicological characterization of the product at recommended doses and no carcinogenic effects, mutagenic effects [46], cutaneous and ocular irritability in rabbits [47], cytotoxicity [48], embryo-toxicity [49], and ecotoxicity effects were observed [50]. The molluscicidal activity remains unchanged for 124 days in a dark vial and sealed at room temperature and 736 days under the same conditions under refrigeration at 10–12 °C.

Nowadays, the molluscicidal effect of *E. milii* was tested against *B. glabrata*, *B. straminea*, *B. tenagophila*, *B. pfeifferi*, *Bulinus* sp., *Pseudosuccinea columella*, *Melanoides tuberculata,* and *Achatina fulica* [51,52,53,54]. The positive results against several intermediate hosts allow one to hypothesize the possibility that *E. milii* latex can also be used in the control of several parasites, such as *S. mansoni*, *Fasciola hepatica*, *Paragonimus westermani*, and *Angiostrongylus* sp. Furthermore, the proprieties of *E. milii* were tested against other invertebrates such as the Diptera *Megaselia scalaris* Loew 1866 and the Nematoda *Heterodera cajanikoshi* 1967 [50,55].

Oliveira-Filho and Paumgartten [56] compared the ecotoxicity of *E. milii* latex to niclosamide against the intermediate hosts *B. glabrata* and *B. tenagophila,* non-schistosomiasis related snails (*Helisoma duryi*, *Pomacea* sp.), and nontarget aquatic organisms such as oligochaete (*Tubifex tubifex*), planktonic crustacea (*Daphnia similis*, *Ceriodaphniadubia*, *Artemia* sp.), fishes (*Daniorerio*, *Poecilia reticulata*), frog tadpoles (*Rana catesbeiana*), bacteria (*Pseudomonas putida* and *Vibrio fischeri)*, algae (*Selenas trumcapricornutum* and *Chlorella vulgaris*), and mosquito larvae *Anopheles albitarsis*, *Aedes aegypti*, *Aedes fluviatilis*). In general, *E. milii* latex, as compared with the reference molluscicide niclosamide, is less toxic to non-target aquatic organisms and presents a higher degree of selectivity toward snails, which are intermediate hosts of *Schistosoma* trematodes.

In the *B. glabrata-S. mansoni* model, the exposition to *E. milli* latex for 24 h at concentrations below 2 mg/L significantly affects reproductive biology, energy stocks, and excretory products of infected and uninfected snails. The physiological changes caused by *E. milli* latex were studied by Mello-Silva et al. [22,38]. After exposition to sub-lethal concentrations an intense reduction in the glycogen reserves on digestive gland is observed of *B. glabrata* [22,57]. In snails infected by *S. mansoni*, a similar mechanism is observed; however, infected snails are more sensitive to the compound and express, in the third week of infection, lower levels of glycogen in the digestive gland and cephalopedial mass [38]. El-Ansary et al. [58] attribute the molluscicidal effect of plants to interference in the glycolytic pathway caused by such compounds. The reduction of compatibility for development of the parasite is due to changes in the activity of hexokinase, glucose phosphate isomerase, and pyruvate kinase, three important glycolytic enzymes. It is important to emphasize that the glycolytic pathway is the most important metabolic pathway for infected snails and is affected both in snails exposed to synthetic and natural compounds.

Mello-Silva et al. [59] observed that the exposition of *S. mansoni*-infected snails to the latex caused exhaustion of alternative sources of energy (total proteins), as well as significant variation in the concentration of nitrogen degradation products. According to the authors, increases in urea level and simultaneous decreases in uric acid levels reflect the disturbance caused by latex poisoning in the snail’s metabolism, especially in those infected. This suggests a change in the excretion pattern, moving from uricotelic to ureotelic. These results reflect the loss of the snails’ ability to regulate their metabolism due to intoxication from *E. milii* latex exposure [59]. This fact corroborates the hypothesis of selective action of *E. milii* latex on the control of *B. glabrata* populations in control actions of schistosomiasis [22].

Since *E. milii* latex is a plausible alternative in the control of schistosomiasis, De-Carvalho et al. [60] analyzed the possible effects of this compound in the other phases of the *S. mansoni* cycle. In graded concentration tests (from 10 to 100 mg/L), the authors did not observe lethal effects on eggs, miracidia, or cercaria. Although the concentration demonstrates no effect on the survival of *S. mansoni*, there is evidence of a positive correlation between exposure time and mortality of miracidia and cercariae [61].

Nevertheless, the toxic effects of the environment on parasitic forms go beyond the analysis of survival and mortality. Augusto et al. [24] studied the viability of the *S. mansoni* cycle after contact of eggs, miracidia, and infected snails (1–8 weeks of infection) with the same latex. According to the authors, the exposure negatively influenced the development of the parasite in the intermediate host with a consequent reduction in the elimination of cercariae in all groups observed. Recently, Augusto et al. [62] demonstrated that transient exposure of cercariae to the latex at doses that do not affect its infectivity has effects 60 days later on the morphology, physiology, and fitness of the adult parasite worms. Using comparative transcriptomics and proteomics approaches, the authors described that the effect of latex on the adult is not due to direct toxicity but it triggers an early change in developmental trajectory and perturbs cell memory, mobility, energy metabolism, and other key pathways, concluding that latex not only has an effect on the snail vector but applies also long-lasting schistosomastatic action.

## 4. Phytochemical Molluscicides and Schistosomiasis: What We Still Need to Learn

On schistosomiasis control, despite the efficacy of mass drug administration (MDA) campaigns in reducing morbidity, a decrease in the transmission of the parasite was not achieved [63]. Treatment of freshwater bodies with molluscicidal compounds is considered an important element in an integrated strategy for transmission control. However, the use of synthetic molluscicides is impeded by the high costs for some countries, there is a demand for inexpensive alternatives such as natural products. In this sense, several plants were tested as a source of potential phytochemical molluscicides [23,64]. In China, where regular MDA programmes are in place, the timing of molluscicide application to reduce snails’ densities is just before the delivery of PZQ. It is important for chemotherapy and snail control teams to be fully integrated because the optimum time for preventive chemotherapy is when there is no risk of reinfection, i.e., when snails have been reduced and transmission is halted. Based on Chinese experience, snail control should be conducted 5–7 weeks before chemotherapy to reduce the transmission potential.

Another important point that should consider before molluscicide application is linked to the cost-effectiveness of the compound to be used [15]. Mollusciciding programmes should take into account the logistical and environmental costs involved in their approaches, such as impact on non-target snails and the wider aquatic ecosystem. It is important to gather as much knowledge as possible about the molluscicide, how and when to apply, and the sensibility of entire fauna in the study area, and every effort should be made to restrict mollusciciding to known disease transmission sites. Places of high species endemicity should be treated extremely cautiously [15].

The availability of modern technologies opens new horizons for understanding the effects of the *E. milii* latex as a molluscicide and as a schistosomastatic (capable of inhibiting the development of schistosomes, while not necessarily killing them otherwise) on a large-scale project. Although some descriptions on the composition of this product can be found in the literature, there is still little information on the structural stability in different geographic regions, through the years and different seasons. This knowledge is essential in large-scale projects in view of variations in lethal power of the latex could affect the effectiveness of control actions, such as for latex supply and maintenance during the occurrence of control actions.

In addition, studies on the phenotypic and physiological effect of latex on naive and infected snails are not uncommon in the literature; however, few studies at the molecular level can be found. Essentially, studies such as those on the immune response system and gene expression are needed to clarify the compatibility factors observed in previous works [24,59,61]. Additionally, phytochemical studies usually show the effect on snails or on a specific parasite stage, leaving aside the complexity of the life cycle and the consequences on multiple steps and over generations. Some of these questions are essential to understand how this compound can be used out of laboratory conditions [24,59,61].

Field tests performed in Comercinho, the municipality of Vale do Jequitinhonha, northeast of Minas Gerais, eliminated the presence of the intermediate host mollusc for the period of three consecutive months [65]. According to the authors, the recolonization of the experimental sites may be related to escape mechanisms and/or the hatching of new spawnings from untreated areas, and this fact suggests that latex applications should be carried out periodically. Schall et al. [40] performed similar tests in different endemic regions of Minas Gerais with interruption of the *S. mansoni* cycle for a period of 14 months. However, both studies lack complementary information about the effects of latex on non-target organisms, on parasitic cycles, and on the physico-chemical properties of field solutions, among others.

In conclusion, an understanding of how *E. milii* latex affects the snail vector and their parasites from a molecular level to field conditions is lacking. On the other hand, this type of treatment could also provide a rationale for the control of schistosomiasis and other parasitosis. Several publications contribute to enforcing the use of *E. milii* latex endemic countries as a cheap alternative or as a complement to mass drug treatment with praziquantel, the only drug available for curing patients (without preventing re-infection).
